# Preparation of Low-Carbon Ferroaluminate Cement with High Iron Red Mud and Multi-Solid Wastes: Mineral Phase Optimization and Hydration Mechanism

**DOI:** 10.3390/ma19173689

**Published:** 2026-08-30

**Authors:** Shanliang Ma, Xiaoming Liu, Zengqi Zhang, Yu Xue, Jianyang Gao

**Affiliations:** 1 State Key Laboratory of Advanced Metallurgy, University of Science and Technology Beijing, Beijing 100083, China; 2 School of Metallurgical and Ecological Engineering, University of Science and Technology Beijing, Beijing 100083, China; d202310154@xs.ustb.edu.cn (S.M.); xy2440236650@163.com (Y.X.); 3 China Aluminum Shandong Co., Ltd., Zibo 255051, China

**Keywords:** ferroaluminate cement, high iron red mud, industrial solid waste, compressive strength, hydration mechanism, mineral phase optimization

## Abstract

The large-scale accumulation of industrial solid wastes has caused serious environmental concerns, while the traditional cement industry urgently requires low-carbon alternatives to reduce CO_2_ emissions. In this study, a solid waste-based ferroaluminate cement (SWFAC) was successfully prepared using high iron red mud (HIRM) and multi-solid wastes. The effects of calcination temperature, holding time, and raw material composition on clinker mineral evolution were systematically investigated, and the relationship among mineral composition, mechanical properties, and hydration behavior was further elucidated. The results demonstrated that increasing the calcination temperature promoted the formation of C_4_A_3_$, C_2_S, and C_4_AF, while highly reactive alumina facilitated clinker formation. The optimized SWFAC clinker exhibited a balanced mineral assemblage with high contents of C_4_A_3_$ and C_4_AF, resulting in excellent mechanical performance. Increasing HIRM content regulated the Fe-bearing phase formation and improved the long-term stability of the cement matrix. Hydration mechanism investigations revealed that the formation of AFt, AFm, AH_3_, and Fe-AFt hydration products facilitated the progressive refinement and densification of the cement matrix, thereby promoting the development and long-term enhancement of compressive strength. This work provides a feasible strategy for the synergistic utilization of industrial solid wastes and the development of low-carbon ferroaluminate cement.

## 1. Introduction

The rapid development of steel, non-ferrous metallurgy, and energy industries has generated large quantities of industrial solid wastes, including red mud (RM), circulating fluidized bed fly ash (CFBFA), desulfurization ash (DSA), and carbide slag (CS). Taking RM as an example, the annual production of RM in China has exceeded 100 million tons, with a cumulative stockpile of more than 1.5 billion tons [1], while its utilization rate remains below 15% [2,3]. Meanwhile, the annual production of fly ash in China has reached several hundred million tons. Among them, CFBFA is difficult to be widely utilized through conventional fly ash-based approaches due to its high contents of free CaO (f-CaO) and SO_3_ [4,5]. These solid wastes are commonly disposed of by stockpiling or landfilling, which not only occupies considerable land resources but also causes environmental risks [6]. On the other hand, the cement industry is a typical energy-intensive and carbon-intensive industry. Cement production contributes approximately 7–8% of global CO_2_ emissions [7,8], mainly originating from limestone decomposition and fuel combustion during clinker production. Therefore, utilizing bulk industrial solid wastes, such as RM and CFBFA, for the preparation of low-carbon cementitious materials can not only alleviate the pressure caused by solid waste accumulation but also provide an effective pathway for carbon emission reduction and raw material substitution in the cement industry.

Ferroaluminate cement (FAC) has been considered a promising low-carbon cementitious material due to its unique clinker mineral composition and excellent engineering properties [9,10]. Compared with ordinary Portland cement, FAC clinker mainly consists of ye’elimite (C_4_A_3_$), belite (C_2_S), and tetracalcium aluminoferrite (C_4_AF) [11,12]. These minerals can generally be formed at lower temperatures than those required for Portland cement clinker, providing FAC with inherent advantages in reducing thermal energy consumption and process-related CO_2_ emissions [13,14]. In addition, FAC typically exhibits high early strength, rapid hardening, slight expansion, good frost resistance, sulfate resistance, and seawater corrosion resistance, making it suitable for emergency repair engineering, marine structures, sulfate-resistant concrete, and precast components [15,16,17]. From the perspective of chemical composition, the FAC clinker system exhibits high compatibility with various industrial solid wastes. High iron red mud (HIRM) is rich in Fe_2_O_3_ and Al_2_O_3_ [18,19], which can provide the primary iron and aluminum sources for the formation of ferroaluminate and calcium sulfoaluminate phases. CFBFA contains abundant SiO_2_, Al_2_O_3_, CaO, and sulfur-bearing components [20], which can participate in the formation of C_2_S and C_4_A_3_$ phases. DSA can provide a stable SO_3_ source, promoting the formation of calcium sulfoaluminate minerals [21]. CS contains a high CaO content and can serve as an alternative calcium source to replace conventional limestone. The synergistic utilization of multiple solid wastes enables the complementary supply of key elements, including Ca, Al, Fe, Si, and S. This approach can not only reduce the consumption of natural mineral resources but also enhance the resource utilization efficiency of industrial solid wastes, thereby achieving the synergistic development of low-carbon cementitious materials and high-value solid waste utilization.

However, the preparation of FAC from HIRM and multi-source solid wastes still faces several critical scientific challenges. First, the occurrence states and reactivity of different components in multi-source solid wastes vary significantly. In particular, aluminum in red mud mainly exists in the form of stable aluminosilicate phases or hydrated minerals, whereas bauxite and industrial alumina exhibit different levels of reactivity. The influence of these differences in alumina source structure on early-stage solid-state reactions and the formation behavior of target minerals, such as C_4_A_3_$ and C_4_AF, remains insufficiently understood [22,23,24]. Second, CaO, Al_2_O_3_, Fe_2_O_3_, SiO_2_, and SO_3_ components in multi-source solid waste systems simultaneously participate in complex solid-state reactions. The clinker phase evolution is governed by the coupled effects of raw material composition, heating process, and holding time [25,26]. However, the mechanisms by which calcination temperature and holding time regulate intermediate phase transformation and target mineral formation in solid waste-based ferroaluminate cement (SWFAC) remain unclear [27,28,29]. Furthermore, previous studies have mainly focused on the utilization of individual solid wastes or relatively simple binary waste systems. For complex multi-source waste systems containing HIRM, CFBFA, DSA, and CS, it remains necessary to clarify how rational regulation of raw material composition can promote the synergistic formation of high-content C_4_A_3_$, C_2_S, and C_4_AF phases [30,31]. Moreover, the underlying relationships among clinker mineral composition, hydration behavior, and mechanical performance require further investigation [32].

In this study, low-carbon SWFAC was prepared using HIRM, CFBFA, DSA, CS, and bauxite (BR) as the main raw materials. The effects of calcination temperature, holding time, and raw material composition on clinker mineral formation were systematically investigated. XRD-Rietveld method was employed to quantitatively analyze the evolution of key phases. Furthermore, the mechanical properties and hydration behavior of the optimized clinker were evaluated to elucidate the relationship between mineral composition and performance development. Different from previous studies mainly focusing on individual solid waste utilization or relatively simple waste systems, this work proposes a synergistic utilization strategy of multi-source industrial solid wastes and reveals the coupling effects of Ca–Al–Fe–Si–S components on ferroaluminate clinker formation. Moreover, the role of Fe derived from high-iron red mud in regulating ferrite phase formation and hydration behavior was further clarified. This study provides a feasible strategy for the synergistic preparation of low-carbon FAC from HIRM and multi-source industrial solid wastes, and offers new insights into the design and application of solid-waste-based cementitious materials.

## 2. Materials and Methods

### 2.1. Raw Materials

**Figure 1:**
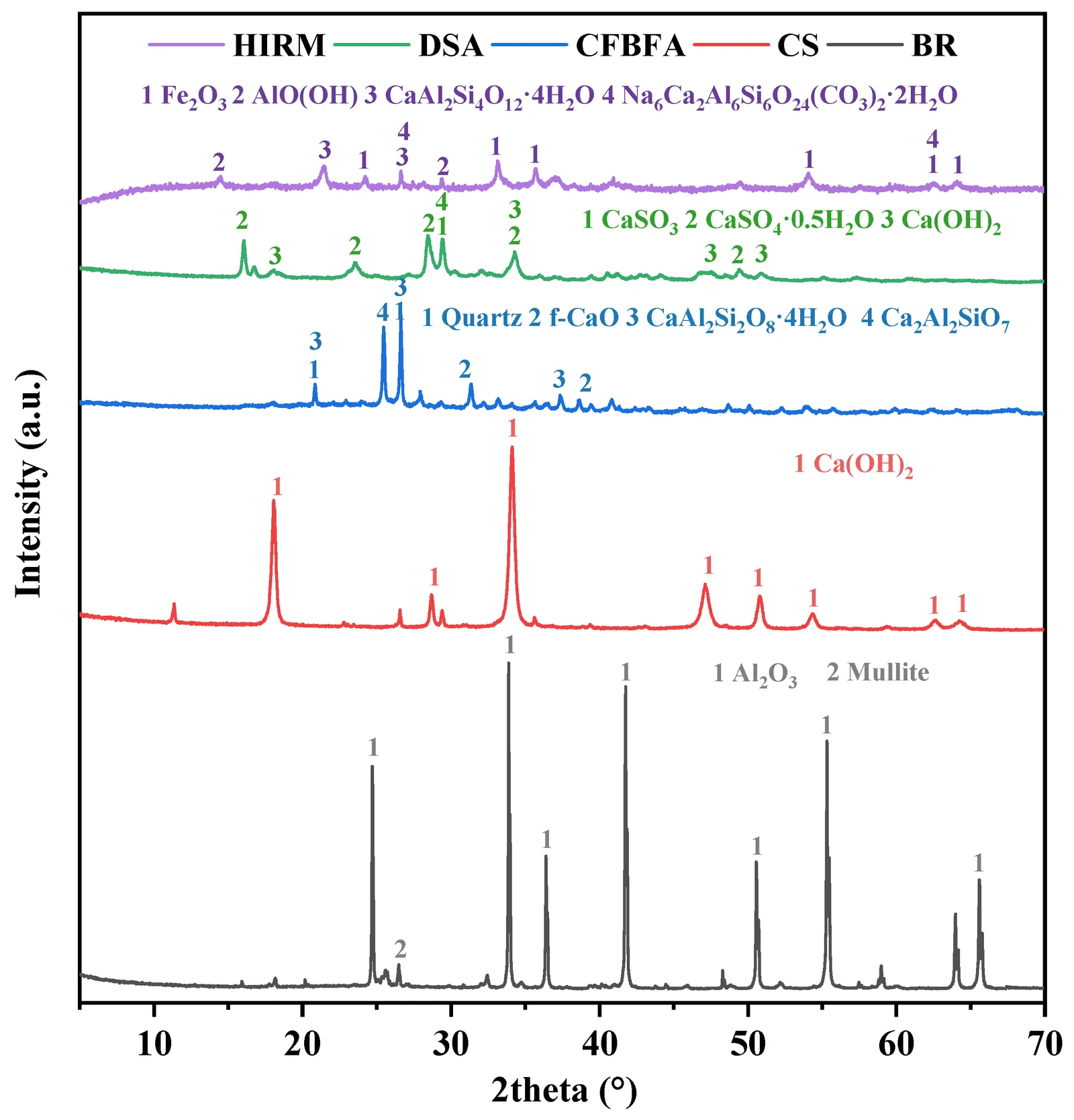
XRD patterns of raw materials [33].

High-iron red mud (HIRM), circulating fluidized bed fly ash (CFBFA), desulfurization ash (DSA), and carbide slag (CS) were obtained from Wenfeng Group Co., Ltd., Tangshan, China. Bauxite residue (BR) was purchased from Henan Borun New Materials Co., Ltd., Ningbo, China. In addition, analytical-grade alumina (Al_2_O_3_) was purchased from Shanghai Macklin Biochemical Co., Ltd., Shanghai, China and used as a high-purity aluminum source for comparison. The chemical compositions of the raw materials were determined using X-ray fluorescence (XRF) spectroscopy and are listed in Table 1. The mineral compositions of the raw materials were identified by X-ray diffraction (XRD) analysis, as shown in Figure 1.

### 2.2. Experimental Design

Based on preliminary experiments, two mix designs were selected for investigating the effects of calcination temperature and holding time, as summarized in Table 2. Both T1 and T2 raw mixtures consisted of HIRM, CFBFA, DSA, and CS as the main raw materials, while differing in the type of alumina source. In the T1 mixture, 15 wt.% BR was incorporated as the alumina source, whereas in the T2 mixture, BR was replaced by 15 wt.% Al_2_O_3_. The CaO, Al_2_O_3_, Fe_2_O_3_, and SO_3_ contents in both formulations were controlled within the compositional range suitable for FAC clinker formation [34]. The raw mixtures were calcined at temperatures ranging from 1000 to 1300 °C with holding times of 30–60 min. The purpose of this comparison was to evaluate the effects of alumina source characteristics, calcination temperature, and holding time on clinker phase formation and structural evolution, providing a basis for optimizing the raw meal composition.

To further investigate the optimal dosage of HIRM and optimize clinker performance, a series of raw meal designs were designed based on the optimized calcination conditions, as listed in Table 3. To maximize solid waste utilization, the proportion of industrial solid wastes was maintained above 80%, while achieving a high-iron and low-calcium clinker composition. All raw mixtures were calcined at 1300 °C for 30 min to investigate the effects of raw material proportion on the formation of key clinker phases.

All raw materials were ground using a ball mill until they passed through a 200-mesh sieve and were then stored for further use. According to the raw meal mix design, the required amounts of each raw material were accurately weighed and thoroughly mixed. The prepared raw meals were homogenized and then mixed with 10 wt.% water. The mixtures were subsequently pressed into disk-shaped pellets (φ 40 mm × 10 mm) under a pressure of 20 MPa. The pressed pellets were dried in an oven at 105 °C for more than 12 h before calcination. The dried raw meal pellets were then placed in a high-temperature furnace for clinker preparation. The calcination procedure was designed as follows: the samples were first heated from room temperature to 1000 °C at a heating rate of 7.5 °C/min, followed by further heating to the target temperature at 5 °C/min and holding for a predetermined time. After the holding stage, the samples were immediately removed from the furnace and rapidly cooled to room temperature using forced air cooling to obtain the clinker. The obtained clinker was subsequently ground using a grinder until the residue retained on a 75 μm square-hole sieve was below 10%. The ground clinker was stored in sealed bags and thoroughly mixed with 15 wt.% gypsum to prepare the cement samples.

### 2.3. Testing and Characterization Methods

#### 2.3.1. Free CaO Content

The free CaO (f-CaO) content in the cement clinker was determined using the ethylene glycol method [35]. Phenolphthalein was used as the indicator, and the extracted Ca^2+^ ions were titrated using a benzoic acid–anhydrous ethanol solution. The f-CaO content was used to evaluate the burnability of the raw meal and the completeness of clinker formation.

#### 2.3.2. Quantitative Phase Analysis

The phase compositions of SWFAC clinker were quantitatively analyzed by XRD combined with internal-standard Rietveld refinement. XRD measurements were performed using a Bruker D8 Advance diffractometer (Bruker AXS, Karlsruhe, Germany) equipped with Cu Kα radiation. The conventional XRD patterns were collected over a 2θ range of 5–70°, while the diffraction data for quantitative phase analysis were collected over a 2θ range of 5–90° and analyzed by Rietveld refinement using GSAS-II software (Version 5799) to quantitatively determine the mineral compositions of the clinker [28,36].

#### 2.3.3. Setting Time and Compressive Strength

Cement pastes and mortars were prepared with a W/B ratio of 0.5 and a B/S ratio of 1:3, respectively. The setting time was measured using a Vicat apparatus according to the Chinese standard GB/T 1346-2024 [37]. The prepared mortar specimens were cast using molds with dimensions of 40 × 40 × 40 mm^3^. After casting, the specimens were cured in a standard curing box at 20 ± 2 °C for 12 h before demolding, and then further cured until the ages of 1, 3, and 28 d. The compressive strength was measured using a mechanical testing machine, and the final results were calculated as the average value of three parallel specimens.

#### 2.3.4. Heat of Hydration Analysis

The heat of hydration was measured using an isothermal calorimeter (I-Cal Ultra, Calmetrix Inc., Arlington, MA, USA). Fresh cement pastes were immediately transferred into sealed sample vials and continuously monitored under an isothermal condition of 25 °C.

#### 2.3.5. Hydration Product Analysis

Hydrated paste samples were collected at different curing ages, and hydration was terminated using anhydrous ethanol. After drying, the samples were ground into powders for XRD analysis to investigate the hydration products. Thermogravimetric (TG, Setline TA-Q200, Lyon, France) analysis was further performed to evaluate the thermal decomposition behavior of hydration products and quantify the variations in hydration products, such as AH3, ettringite, and C-S-H gels.

#### 2.3.6. Microstructural Analysis

The microstructures of the clinker and hydration products were examined using a field-emission scanning electron microscope (FE-SEM, Apreo C, Thermo Fisher Scientific, Waltham, MA, USA). To further evaluate the distribution of mineral phases, polished samples were analyzed using backscattered electron (BSE) imaging.

## 3. Results and Discussion

### 3.1. Calcination Condition Optimization

#### 3.1.1. Thermal Decomposition and Phase Transformation of Raw Materials

**Figure 2:**
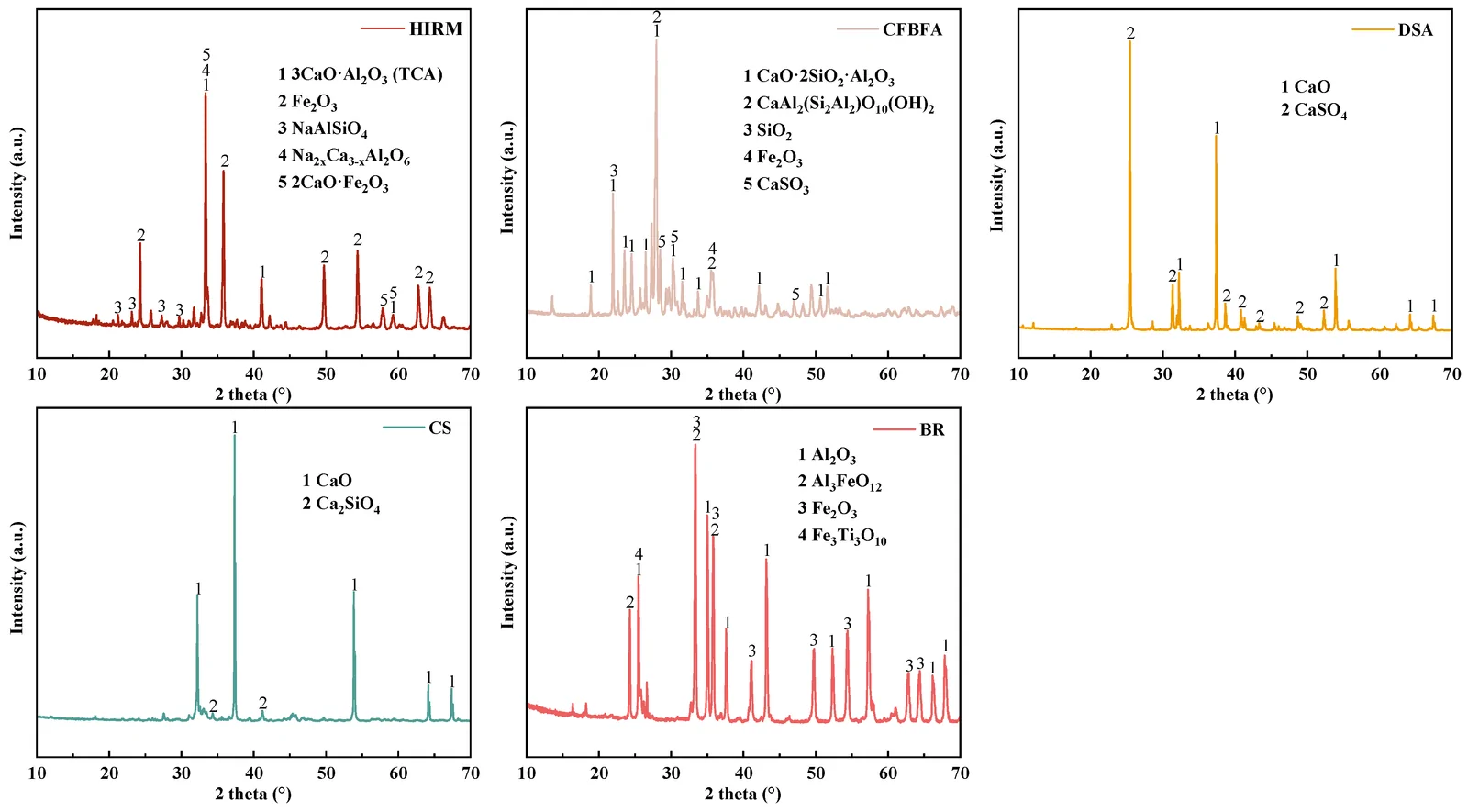
Thermal decomposition and phase transformation characteristics of raw materials calcined at 1200 °C.

Figure 2 shows the thermal decomposition and phase transformation characteristics of the raw materials calcined at 1200 °C. The hydrated minerals in red mud, such as boehmite and hydrogarnet, were completely decomposed and reacted with CaO in the system to form precursor phases, including tricalcium aluminate (TCA) and calcium ferrite (CaO·Fe_2_O_3_). This indicates that red mud can effectively provide reactive Fe_2_O_3_ and Al_2_O_3_ sources at this temperature. For CFBFA, partial SiO_2_ and Al_2_O_3_ reacted with CaO to form calcium aluminosilicate phases, while CaSO_3_ remained relatively stable, suggesting that the sulfur source was not completely oxidized at this stage. DSA was almost completely transformed into CaSO_4_ at 1200 °C, providing a stable SO_3_ source for the formation of C_4_A_3_$. CS underwent complete decomposition of Ca(OH)_2_ into highly reactive CaO. BR underwent dehydration and phase transformation, resulting in the formation of Al_2_O_3_ and providing a stable Al source. Overall, these results demonstrate that the main mineral components in the solid wastes underwent essential decomposition and precursor phase formation at 1200 °C. At this temperature, the decomposition of unstable phases and the generation of reactive intermediate phases were sufficiently achieved, indicating that the raw material system had undergone substantial mineral transformation. Therefore, 1200 °C was selected as a representative temperature to establish the initial mineral evolution characteristics of the raw materials, providing a suitable basis for further investigation of FAC clinker phase formation.

#### 3.1.2. Calcination Temperature Optimization

**Figure 3:**
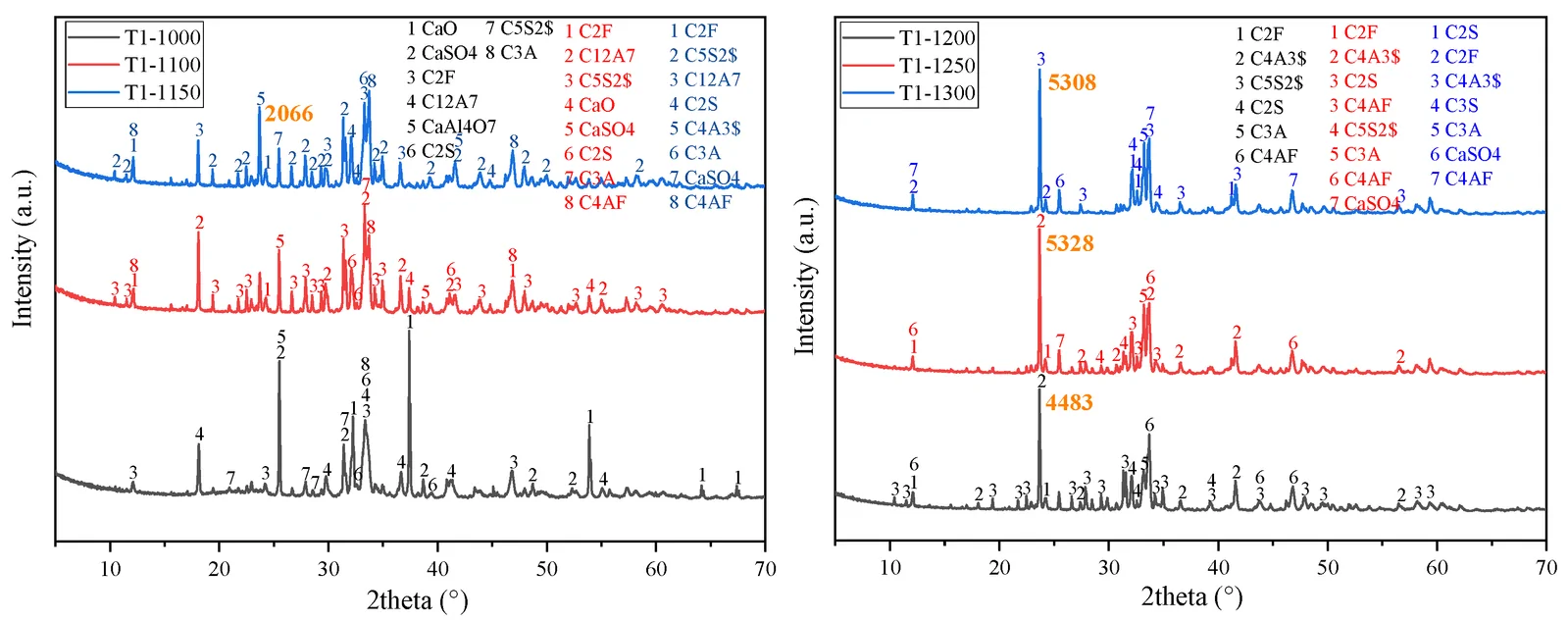
Mineral phase transformation of T1 sample at different calcination temperatures.

Figure 3 illustrates the phase evolution of the T1 sample during calcination at different temperatures. The mineralogical transformation of the raw meal exhibited distinct stage characteristics. At 1000 °C, the clinker system was mainly composed of free CaO, CaSO_4_, and intermediate aluminate phases, such as C_12_A_7_ and CaAl_4_O_7_. The target clinker phases had not yet formed in large quantities, indicating that this stage was mainly associated with the decomposition of carbonates, hydroxides, and hydrated phases in the solid wastes, accompanied by the formation of primary aluminate reaction products. When the temperature increased to 1100–1150 °C, C_2_S and C_2_F phases began to appear. Meanwhile, a considerable number of intermediate phases remained, suggesting that the system entered a rapid solid-state reaction stage, although the clinker phase assemblage had not yet reached a stable state. With further increasing the temperature to 1200 °C, the characteristic peaks of C_4_A_3_$ became clearly detectable. Meanwhile, the diffraction intensities of C_2_S and C_2_F increased significantly, whereas the peaks corresponding to f-CaO and some intermediate phases weakened. These results indicate that 1200 °C provided suitable thermodynamic and kinetic conditions for the formation of the main ferroaluminate clinker phases. Upon further heating to 1250–1300 °C, C_4_A_3_$, C_2_S, and C_4_AF became the dominant mineral phases. Their diffraction peaks reached the highest intensities, and the diffraction patterns became relatively stable, with only a small amount of residual CaSO_4_ detected. This suggests that the clinker formation reaction was highly promoted within this temperature range, resulting in a phase assemblage close to the designed composition.

**Figure 4:**
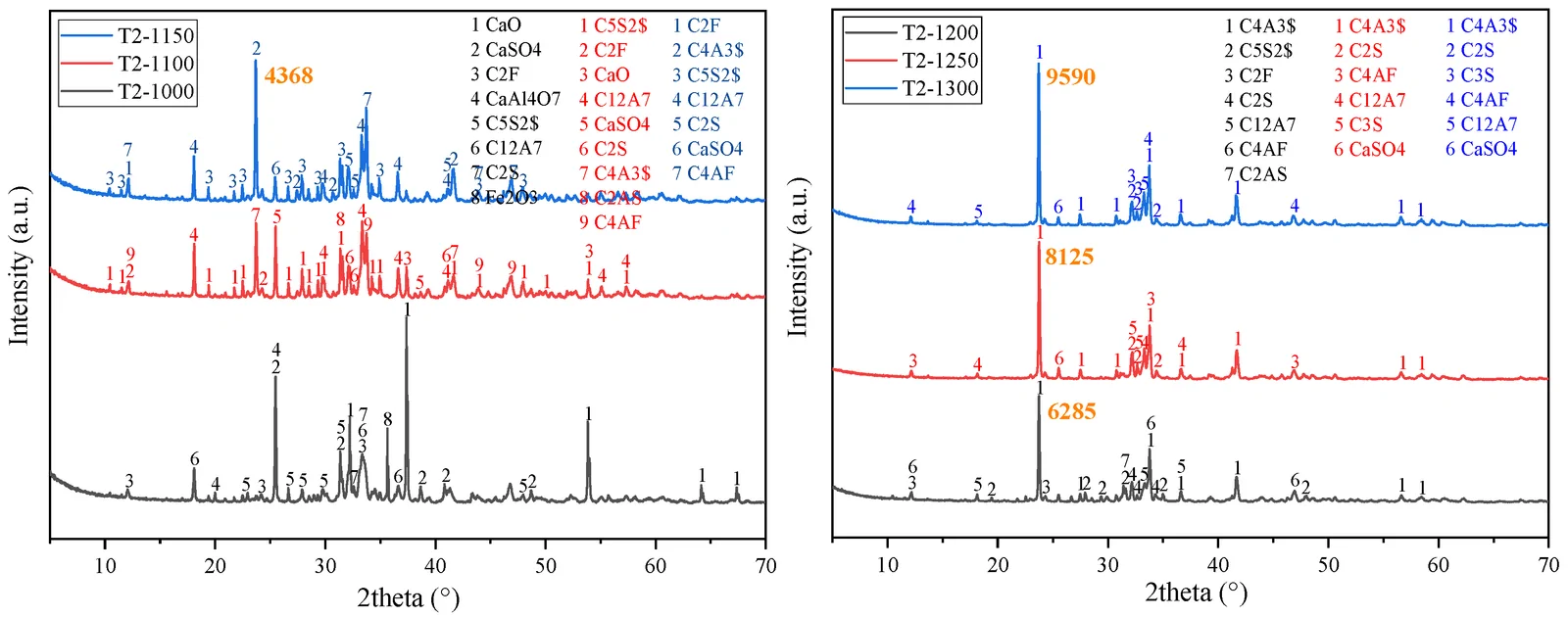
Mineral phase transformation of T2 sample at different calcination temperatures.

As shown in Figure 4, the phase evolution behavior of the T2 sample at different calcination temperatures exhibited similar staged characteristics to that of T1. However, due to the replacement of bauxite residue with highly reactive alumina as the aluminum source, the phase formation process in T2 proceeded more rapidly. At 1000–1100 °C, C_12_A_7_, C_2_F, and several early-stage aluminate intermediate phases appeared earlier and exhibited higher diffraction intensities in T2 than in T1, indicating that reactive Al_2_O_3_ could participate more effectively in solid-state reactions. When the temperature increased to 1150–1200 °C, distinct characteristic peaks of the target phase C_4_A_3_$ were observed in T2, whereas T1 only showed the initial formation of this phase within the same temperature range. This difference suggests that the solid-state reactions among alumina, sulfate, and calcium species were kinetically more favorable in the T2 system. At 1250–1300 °C, the diffraction intensities of C_4_A_3_$, C_4_AF, and C_2_S in T2 were considerably higher than those in T1, accompanied by a lower f-CaO content. These results indicate that using Al_2_O_3_ as the aluminum source promoted clinker formation and improved the reaction degree of the raw meal. Consequently, the T2 system exhibited a greater ability to stably form the major FAC clinker phases over a relatively broad temperature range.

**Figure 5:**
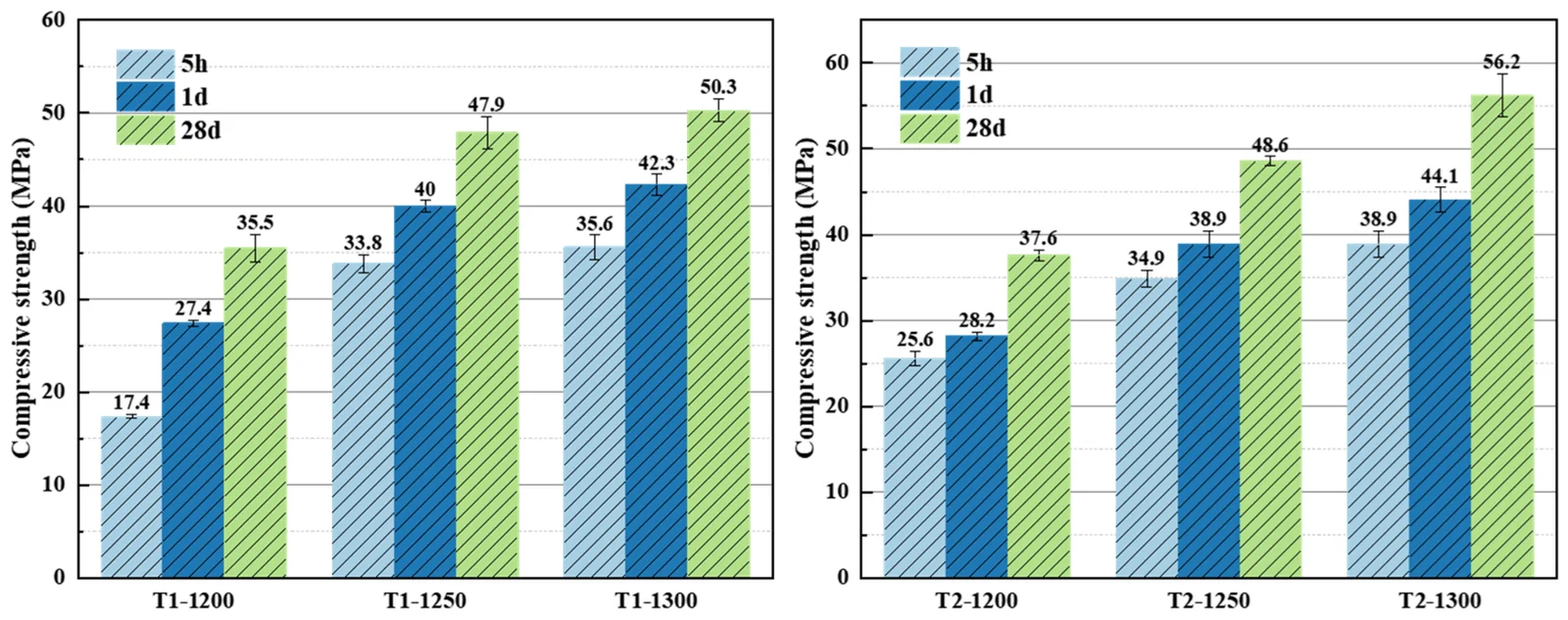
Compressive strength of SWFAC sample at different calcination temperatures.

Figure 5 presents the compressive strength development of SWFAC prepared at different calcination temperatures. It can be observed that the 1, 3, and 28 d compressive strengths of both T1 and T2 samples increased markedly with increasing calcination temperature. For the T1 system, the clinker calcined at 1200 °C exhibited relatively low compressive strengths of 17.4, 27.4, and 35.5 MPa at 1, 3, and 28 d, respectively. When the calcination temperature increased to 1250 °C, the 1 and 3 d compressive strengths increased significantly to 33.8 and 40.0 MPa, respectively, while the 28 d strength further increased to 47.9 MPa, representing an improvement of more than 35%. This result indicates that calcination at 1250 °C promoted the formation of relatively complete clinker mineral phases, including C_4_A_3_$, C_4_AF, and C_2_S. With a further increase in temperature to 1300 °C, the 1, 3, and 28 d compressive strengths of T1 reached 35.6, 42.3, and 50.3 MPa, respectively. Although the strength improvement was less pronounced compared with that observed from 1200 to 1250 °C, the overall mechanical performance remained stable, suggesting that T1 clinker achieved an optimized clinkerization state within the temperature range of 1250–1300 °C. In comparison, T2 exhibited higher compressive strengths than T1 at all curing ages. This improvement was closely associated with the use of highly reactive alumina, which facilitated a more sufficient reaction of the aluminum source and promoted the formation of clinker minerals [38].

#### 3.1.3. Holding Time Optimization

**Figure 6:**
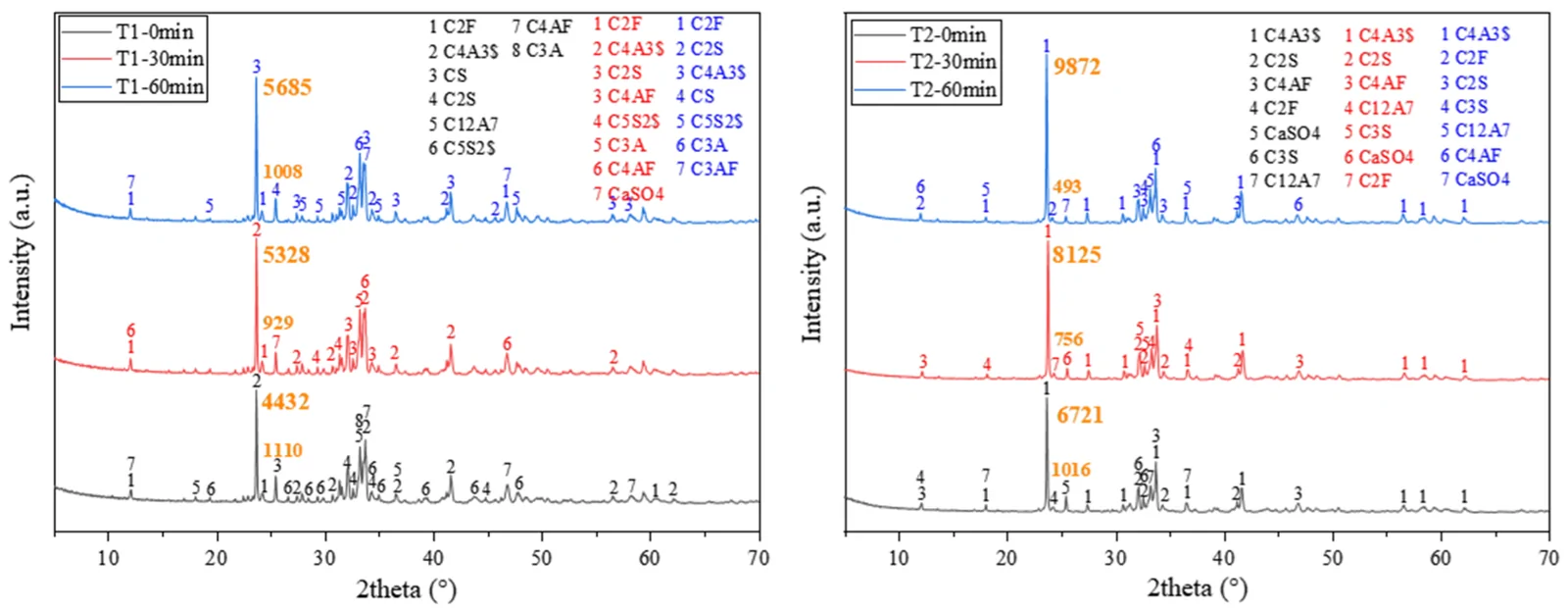
Mineral phase transformation of samples T1 and T2 at different holding times.

Figure 6 shows the phase transformation of T1 and T2 samples calcined at 1250 °C with different holding times. For the T1 system, a considerable number of intermediate phases, including C_12_A_7_, C_2_F, and C_2_S, remained in the clinker at 0 min. The diffraction intensities of these phases were higher than that of the target phase C_4_A_3_$, and a certain amount of residual CaSO_4_ was also detected, indicating that the solid-state reactions were still incomplete at this stage. With increasing holding time to 30 min, the characteristic peaks of C_4_A_3_$, C_4_AF, and C_2_S were significantly enhanced, whereas the intermediate phases, such as C_12_A_7_ and CaSO_4_, together with f-CaO, gradually decreased. This suggests that the formation rate of the key clinker phases was accelerated and the system gradually approached a stable FAC clinker composition. When the holding time was further extended to 60 min, the diffraction intensities of C_4_A_3_$ and C_4_AF in the T1 sample reached their maximum values. This indicates that the solid-state reactions within the clinker were highly promoted and the phase assemblage became relatively stable. In comparison, the T2 system exhibited a faster phase formation process due to the use of highly reactive alumina as the aluminum source. At 0 min, the T2 sample already showed distinct characteristic peaks of C_4_A_3_$, with higher peak intensities than those of the corresponding T1 sample at the same holding time. Meanwhile, a lower amount of residual CaSO_4_ was observed, suggesting that more SO_3_ participated in the formation of calcium sulfoaluminate phases. After 30–60 min of holding, the diffraction intensities of C_4_A_3_$, C_4_AF, and C_2_S in the T2 clinker further increased, exhibiting higher crystallinity and a greater degree of reaction compared with T1. Meanwhile, the intermediate phases were almost completely eliminated.

**Figure 7:**
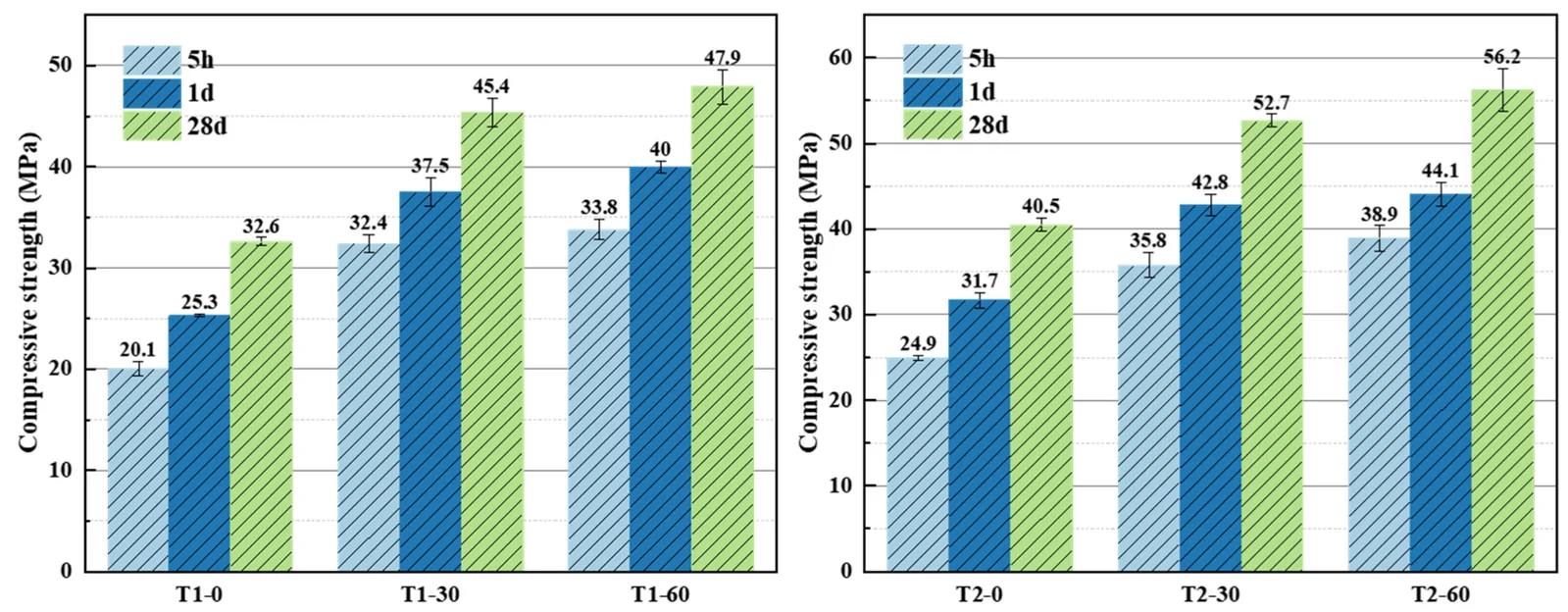
Compressive strength of SWFAC clinker at different holding times.

Figure 7 presents the compressive strength of SWFAC clinkers obtained after different holding times. For the T1 system, the clinker without holding treatment at 1250 °C exhibited relatively poor mechanical performance, with the lowest 1, 3, and 28 d compressive strengths among all T1 samples. This behavior was mainly attributed to the presence of a considerable amount of incompletely reacted intermediate phases, such as C_12_A_7_ and CaSO_4_, resulting in a less compact clinker structure and insufficient early-age strength [39,40]. When the holding time was extended to 30 min, the 1 and 3 d compressive strengths of T1 increased significantly, accompanied by a substantial improvement in 28 d strength. This indicates that prolonged thermal treatment promoted the formation of major clinker phases, including C_4_A_3_$, C_4_AF, and C_2_S, which gradually became the dominant mineral components. With further extension of the holding time to 60 min, the mechanical properties of T1 reached the highest level, and the 28 d compressive strength achieved the maximum value within this group. In contrast, the T2 clinker exhibited much lower sensitivity to holding time due to the use of highly reactive alumina, which accelerated clinker phase formation. The 1, 3, and 28 d compressive strengths of T2 were significantly higher than those of T1 at all holding time periods, indicating that the T2 system possessed enhanced mineral formation capability.

#### 3.1.4. Burnability of Raw Meal

**Figure 8:**
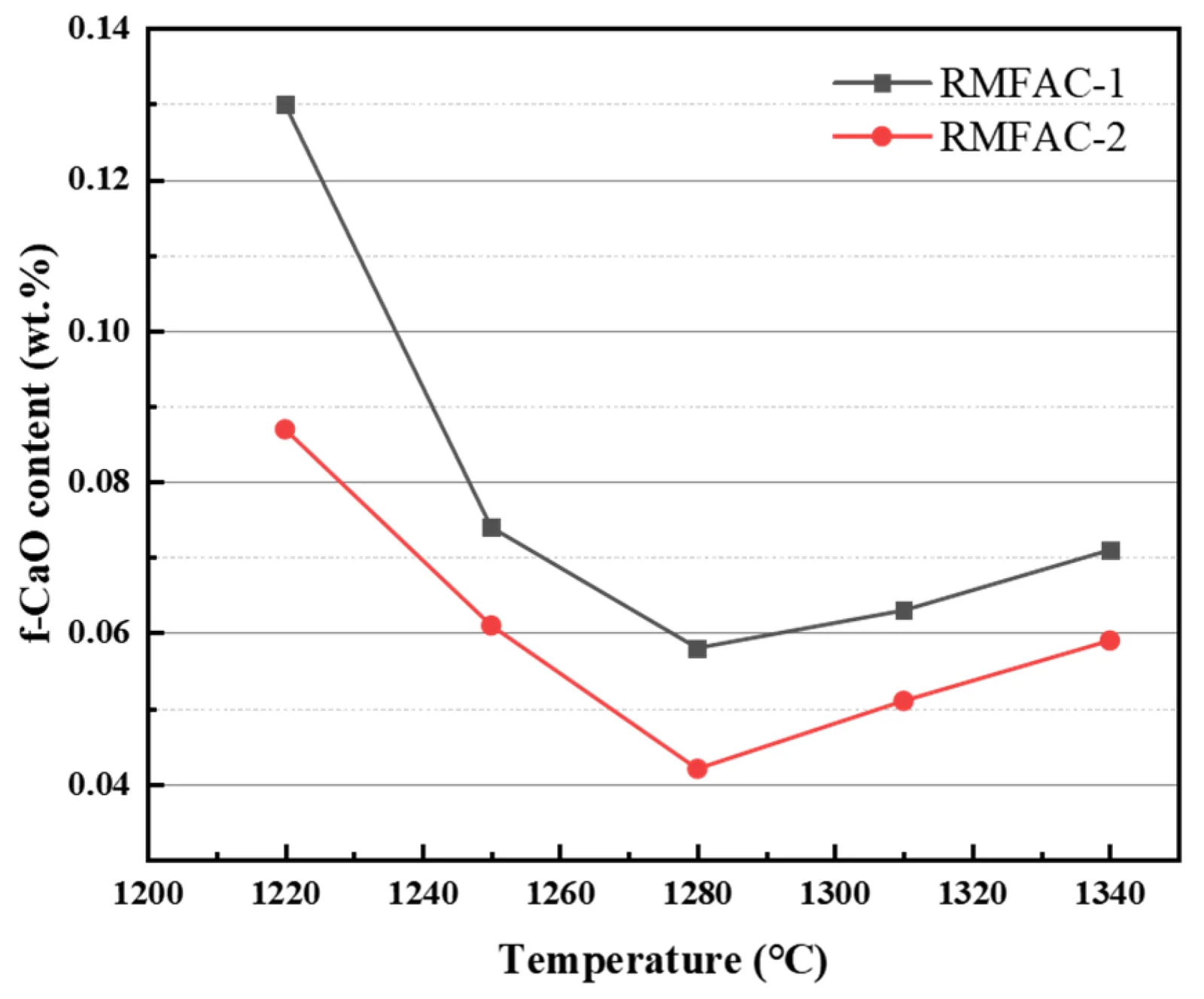
Free CaO content in SWFAC clinker at different calcination temperatures.

The burnability of raw meal is an important indicator for cement clinker production, reflecting the difficulty of converting raw meal into clinker minerals during calcination. This characteristic is commonly evaluated by measuring the f-CaO content in clinker obtained at different calcination temperatures. The f-CaO contents of SWFAC clinkers prepared at different calcination temperatures are shown in Figure 8. The burnability curves of all SWFAC raw meals exhibited a similar trend, with the f-CaO content initially decreasing and then increasing with increasing calcination temperature. Within the temperature range of 1250–1300 °C, the f-CaO contents remained relatively low, indicating that CaO had sufficiently reacted with Al_2_O_3_, Fe_2_O_3_, SiO_2_, and other mineral components. At this stage, the clinker formation process was mainly associated with crystal structure evolution and mineral phase development. Specifically, from 1200 to 1300 °C, the increase in temperature promoted the participation of f-CaO in solid-state reactions, thereby facilitating the formation of effective clinker phases. However, when the temperature reached 1300 °C, the f-CaO content showed an increasing tendency, which was mainly attributed to the partial decomposition of C_4_A_3_$ at elevated temperatures.

### 3.2. Mineral Phase Optimization

#### 3.2.1. Mix Design and Mineral Optimization

Table 4 presents the quantitative phase composition results. As shown, M1 and M3 exhibited higher C_4_A_3_$ contents than M2. The C_4_A_3_$ contents of M1 and M3 reached approximately 34–36 wt.%, which were significantly higher than that of M2 (27.63 wt.%). In contrast, the CFBFA content in M2 was increased to 10 wt.%, resulting in a higher proportion of SiO_2_ and relatively inert components in the system. Consequently, part of the CaO was consumed in the formation of C_2_S, which suppressed the formation of C_4_A_3_$ [41]. As a result, the C_2_S content increased substantially to 43.44 wt.%. The variation in C_4_AF content further supports this trend. M5 exhibited the highest C_4_AF content (26.71 wt.%) and the lowest C_4_A_3_$ content (25.77 wt.%) among the four formulations, indicating that a higher red mud content favored the incorporation of Fe_2_O_3_ into ferrite phases and promoted the formation of iron-bearing minerals [26]. However, it also inhibited the formation of C_4_A_3_$, thereby reducing the rate of early hydration reactions. M4 and M6 were not included in the quantitative phase analysis because they were mainly used for preliminary composition optimization, and their phase evolution trends were comparable to those of the selected representative formulations.

**Figure 9:**
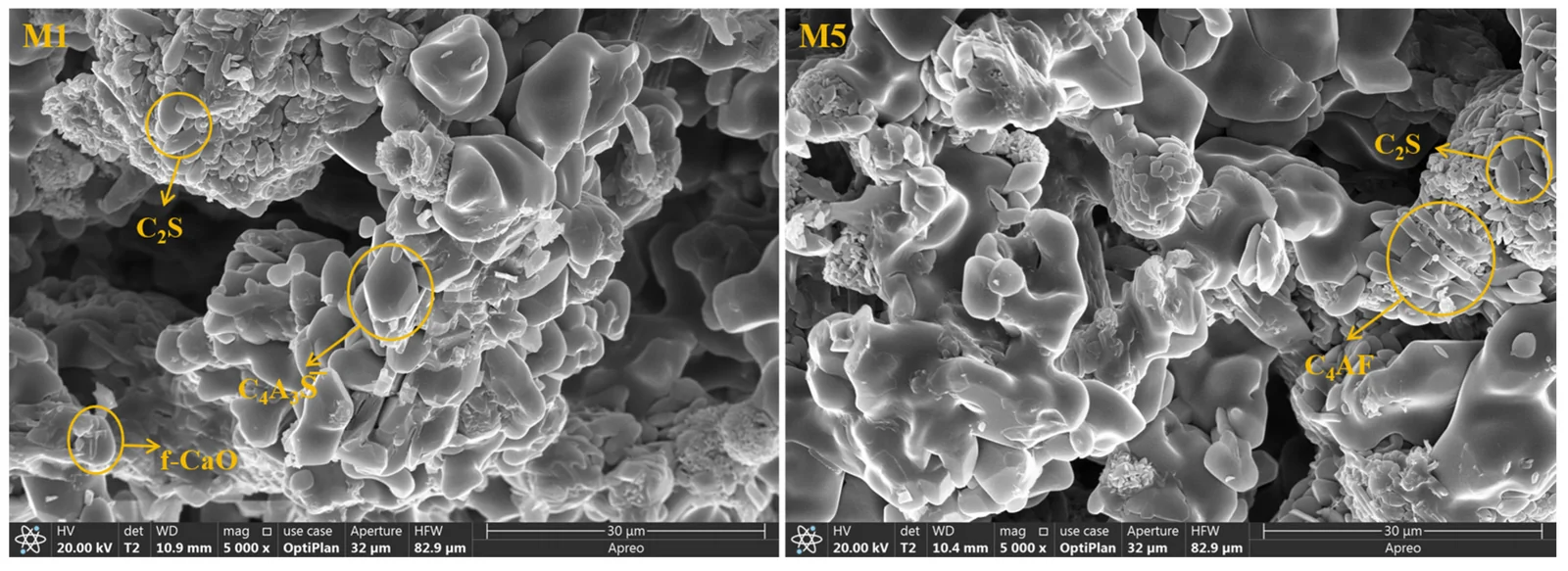
Microscopic morphology of SWFAC clinker.

**Figure 10:**
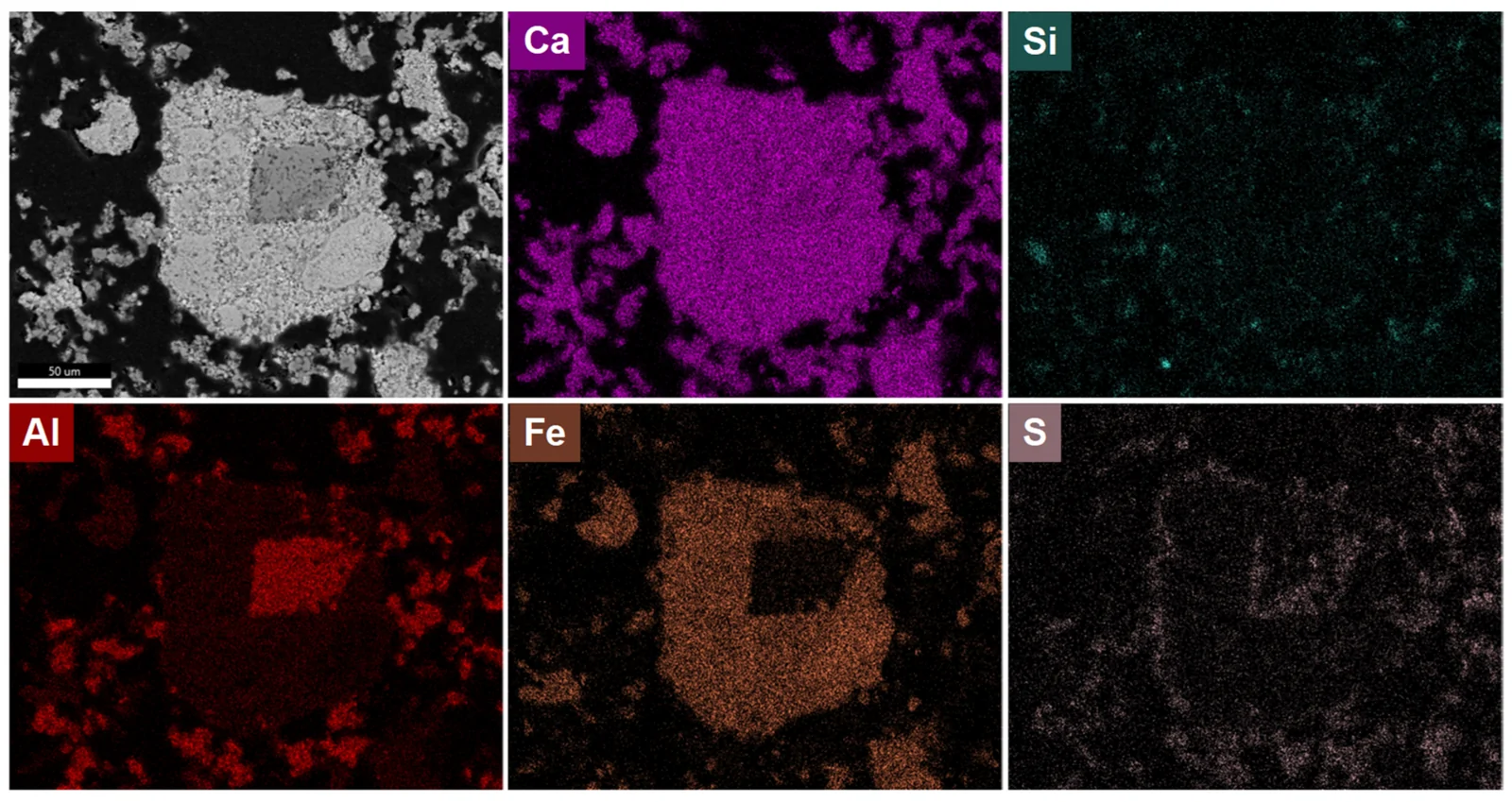
Typical BSE image of SWFAC clinker.

The SEM images of the SWFAC clinker are shown in Figure 9. The C_4_A_3_$ crystals typically exhibited polyhedral and prismatic morphologies with well-defined edges [42], whereas f-CaO crystals were mainly observed as radially distributed needle-like or rod-like structures. In addition, C_2_S crystals showed irregular pebble-like morphologies with varying particle sizes, while C_4_AF crystals generally appeared as elongated columnar structures [43]. These observations indicate that the SWFAC clinker obtained under the optimized calcination regime exhibited good crystal development, clear crystal boundaries, and a relatively uniform phase distribution. Figure 10 presents typical BSE images of the clinker sample. The SWFAC clinker exhibited distinct phase distribution characteristics in the BSE images. Combined with the elemental mapping results for Ca, Al, Fe, Si, and S, Ca was found to be highly concentrated in the major regions of the clinker, indicating the presence of calcium-rich phases such as C_4_A_3_$ and C_2_S. The distributions of Al and Fe generally coincided with the particle contours, suggesting that ferrite phases and calcium sulfoaluminate phases were continuously distributed throughout the clinker matrix. In contrast, Si exhibited a more dispersed distribution, which was mainly associated with the C_2_S phase. Sulfur showed localized enrichment, indicating that it predominantly participated in the formation of C_4_A_3_$. Overall, the major elements exhibited a certain degree of spatial correlation, indicating that the target clinker phases were well developed and uniformly distributed within the system.

#### 3.2.2. Compressive Strength and Setting Time

**Figure 11:**
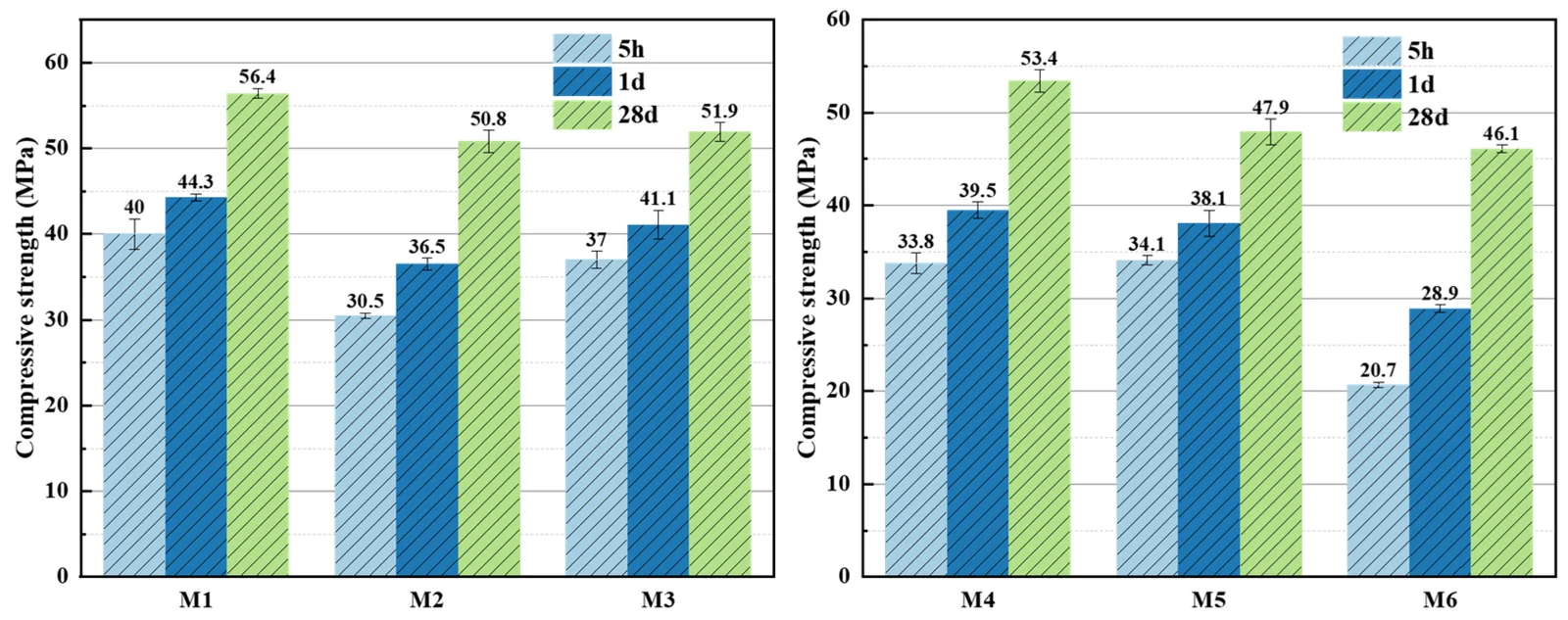
Compressive strength of different SWFAC samples.

Figure 11 presents the relationship between clinker phase composition and compressive strength. Owing to their higher contents of C_4_A_3_$ and C_4_AF, M1 and M3 exhibited the highest contents of effective clinker phases and therefore showed significantly higher compressive strengths at 1, 3, and 28 d than M2. In particular, M3 demonstrated the most pronounced advantage in early-age strength, indicating that the synergistic effect of C_4_A_3_$ and C_4_AF was fully developed, resulting in rapid hydration reactions and the formation of a dense hydration product structure. In contrast, the relatively high C_2_S content in M2 led to lower early-age strength. Although its 28 d strength increased to some extent, it remained lower than those of M1 and M3, suggesting that this formulation was less favorable for obtaining high contents of early-strength clinker phases. As the red mud content increased from 15% to 20% and 25%, the amount of reactive Al_2_O_3_ available in the system decreased slightly, resulting in a moderate reduction in the C_4_A_3_$ content compared with M3. Nevertheless, the overall phase assemblage remained stable, and the proportions of C_4_A_3_$, C_4_AF, and C_2_S remained well balanced without obvious phase imbalance. These results indicate that a red mud content of 20–25 wt.% is still within an acceptable compositional range for maintaining the clinker phase structure.

**Figure 12:**
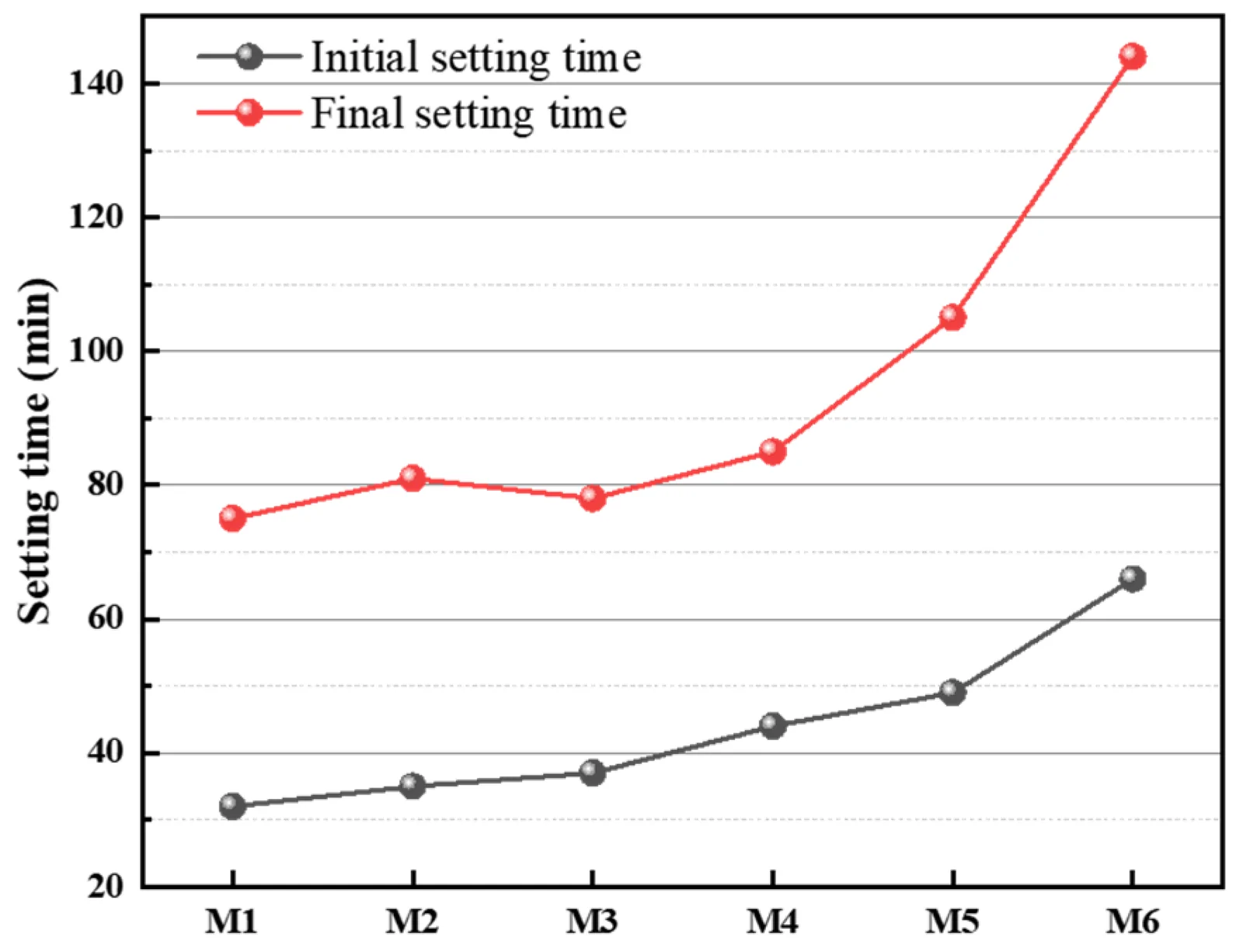
Setting time of different SWFAC samples.

Figure 12 shows the setting times of different SWFAC samples. In terms of the variation trend, increasing the red mud content slightly reduced the C_4_A_3_$ content while increasing the C_4_AF content, thereby decreasing the early hydration heat release rate to some extent. As a result, the initial setting times of M3, M4, and M5 were moderately longer than those of M1 and M2, which is beneficial for alleviating the excessively rapid setting behavior commonly observed in conventional FAC. In particular, M4 and M5 exhibited more desirable setting characteristics, with initial setting times of approximately 35–45 min and final setting times of approximately 80–110 min. These values not only fully satisfy the Chinese national standard requirements (GB/T 45920-2025) but also provide a wider operating window for on-site construction and pumping applications.

### 3.3. Hydration Mechanism

#### 3.3.1. Heat of Hydration

**Figure 13:**
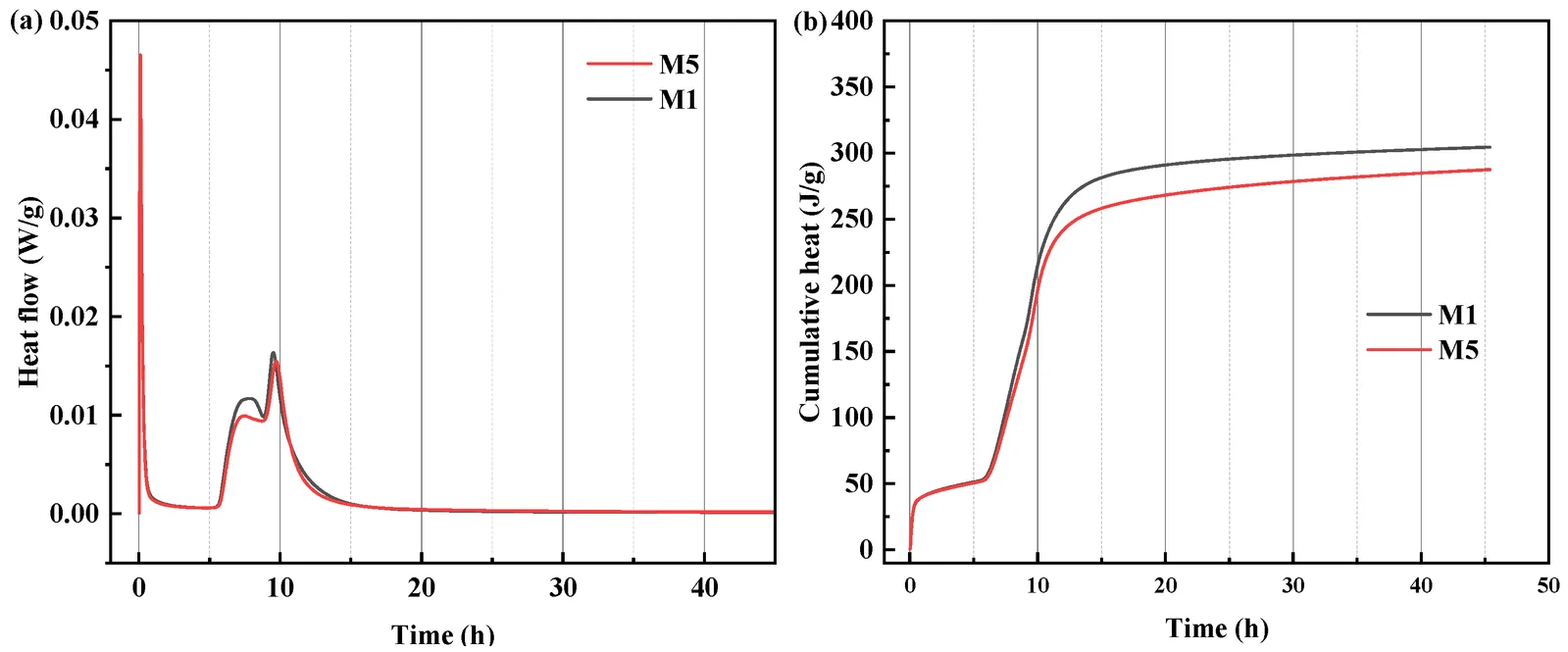
Hydration heat curves of SWFAC: (**a**) Heat flow; (**b**) Cumulative heat.

Figure 13 presents the heat-flow and cumulative-heat curves of the SWFAC samples. Both samples exhibited a pronounced initial exothermic peak immediately after contact with water, which was mainly associated with particle wetting, rapid dissolution of soluble sulfate species, and the release of Ca^2+^, Al-bearing, and other reactive ions from the clinker surface. A broad shoulder followed by a distinct main hydration peak was observed at approximately 6–10 h. The shoulder was primarily attributed to the rapid reaction of C_4_A_3_$ with gypsum and water to form ettringite and AH_3_ [44], whereas the subsequent main peak was related to the continued hydration of the remaining calcium sulfoaluminate, ferrite, and silicate phases. Compared with M5, M1 exhibited higher shoulder and main-peak intensities, indicating a higher early-age hydration rate. This difference can be mainly attributed to the relatively higher proportion of rapidly reactive C_4_A_3_$ in M1. In contrast, the increased HIRM content in M5 reduced the amount of available reactive Al_2_O_3_ and increased the proportion of Fe-bearing phases, particularly C_4_AF, whose hydration proceeded more slowly than that of C_4_A_3_$. The cumulative-heat curves further confirmed this difference. The cumulative heat released by M1 remained higher than that of M5 throughout the monitored period and reached approximately 302 J/g at 45 h, compared with approximately 286 J/g for M5. This result is consistent with the longer setting time, lower early compressive strength, and relatively loose 1 d microstructure of the high-iron sample. Nevertheless, the heat flow of M5 remained continuous during the deceleration stage, suggesting that the Fe-rich clinker phases and C_2_S continued to hydrate at later ages, which contributed to the gradual formation of hydration products and the subsequent densification of the matrix.

#### 3.3.2. Hydration Products

**Figure 14:**
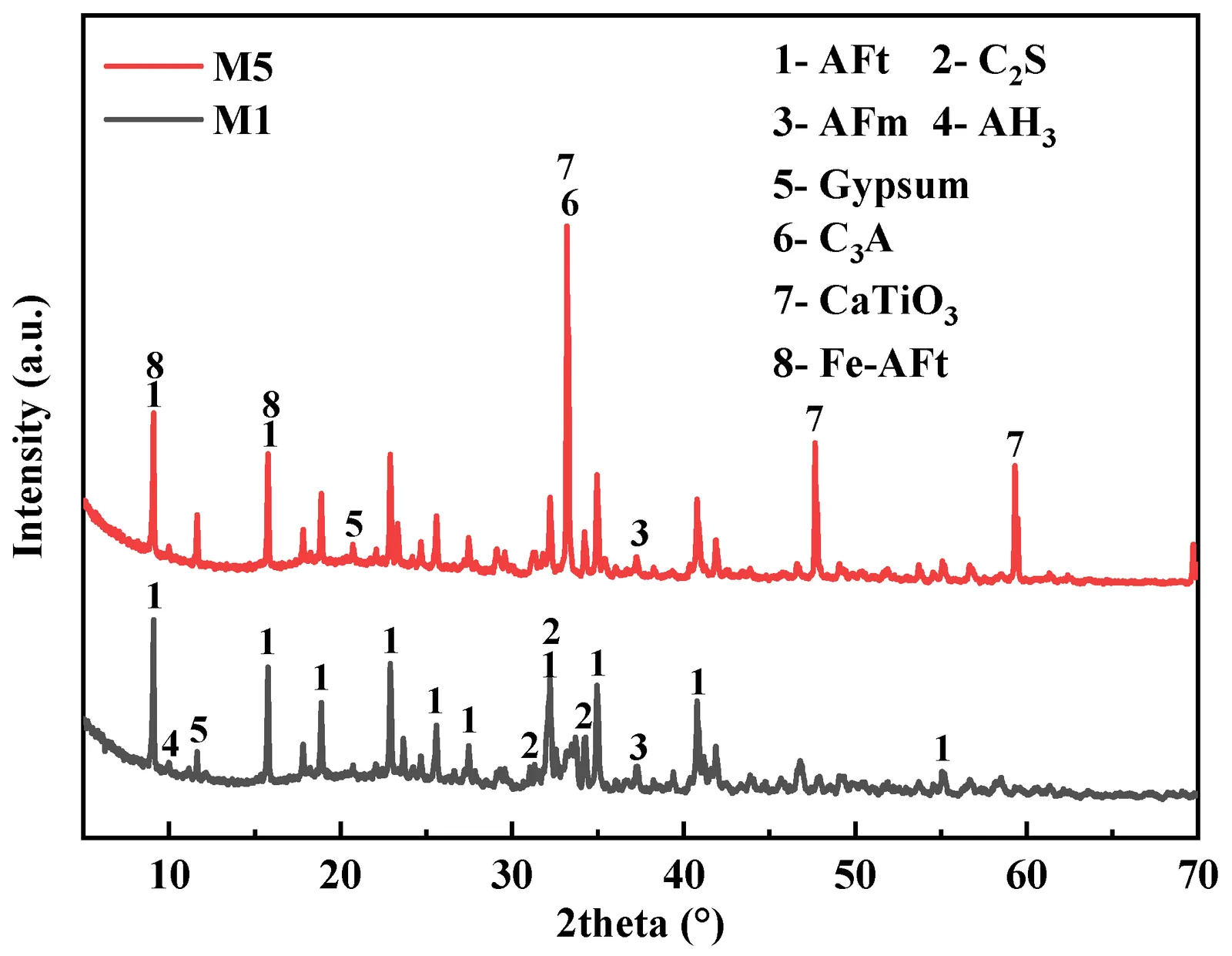
XRD analysis results of SWFAC samples cured for 28 d.

Figure 14 presents the XRD patterns of M1 and M5 samples after 28 d of hydration. Both samples exhibited typical hydration products of ferroaluminate cement, including ettringite (AFt), monosulfate (AFm), and aluminum hydroxide (AH_3_), indicating that the main clinker phases underwent continuous hydration reactions during curing. Meanwhile, residual C_2_S peaks were still detected in both samples, suggesting that the silicate phase was not completely hydrated and continued to contribute to later-age strength development. Compared with M5, the M1 sample exhibited stronger diffraction peaks corresponding to AFt and AH_3_, indicating a higher degree of hydration of the calcium sulfoaluminate phase. This result agrees well with the hydration calorimetry results, where M1 showed a higher hydration heat release rate and cumulative heat than M5. The higher C_4_A_3_$ content in M1 provided more reactive Al-containing phases, which promoted the rapid formation of AFt and AH_3_ during hydration, thereby contributing to the higher early-age strength. In contrast, the M5 sample exhibited characteristic peaks associated with Fe-containing ettringite (Fe-AFt). The formation of Fe-AFt demonstrates that the increased iron content introduced by high-iron red mud actively participated in the hydration process rather than acting merely as an inert component. The substitution of Fe^3+^ for Al^3+^ in the ettringite structure provided an additional pathway for incorporating iron into the hydration matrix. The specific formation processes of the main hydration products are shown in Equations (1)–(5) [33,45]:

(1) $$C_{4}A_{3}\$+2C\bar{S}H_{2}+34H\to C_{6}AS_{3}H_{32}(AFt)+2AH_{3}$$

(2) $$C_{4}A_{3}\$+18H\to C_{4}ASH_{12}(AFm)+2AH_{3}$$

(3) $$C_{2}S+H\to C-S-H\;gels$$

(4) $$2C_{4}AF+14H⟶C_{2}(A,F)H_{8}+C_{3}(A,F)H_{6}+Fe(OH{)}_{3}$$

(5) $$\begin{array}{c} C_{4}AF+3C\$H_{2}+30H\to C_{6}(A,F){\$}_{3}H_{32}\left(Fe-AFt\right)+{(A,F)H}_{3}+CH \end{array}$$

**Figure 15:**
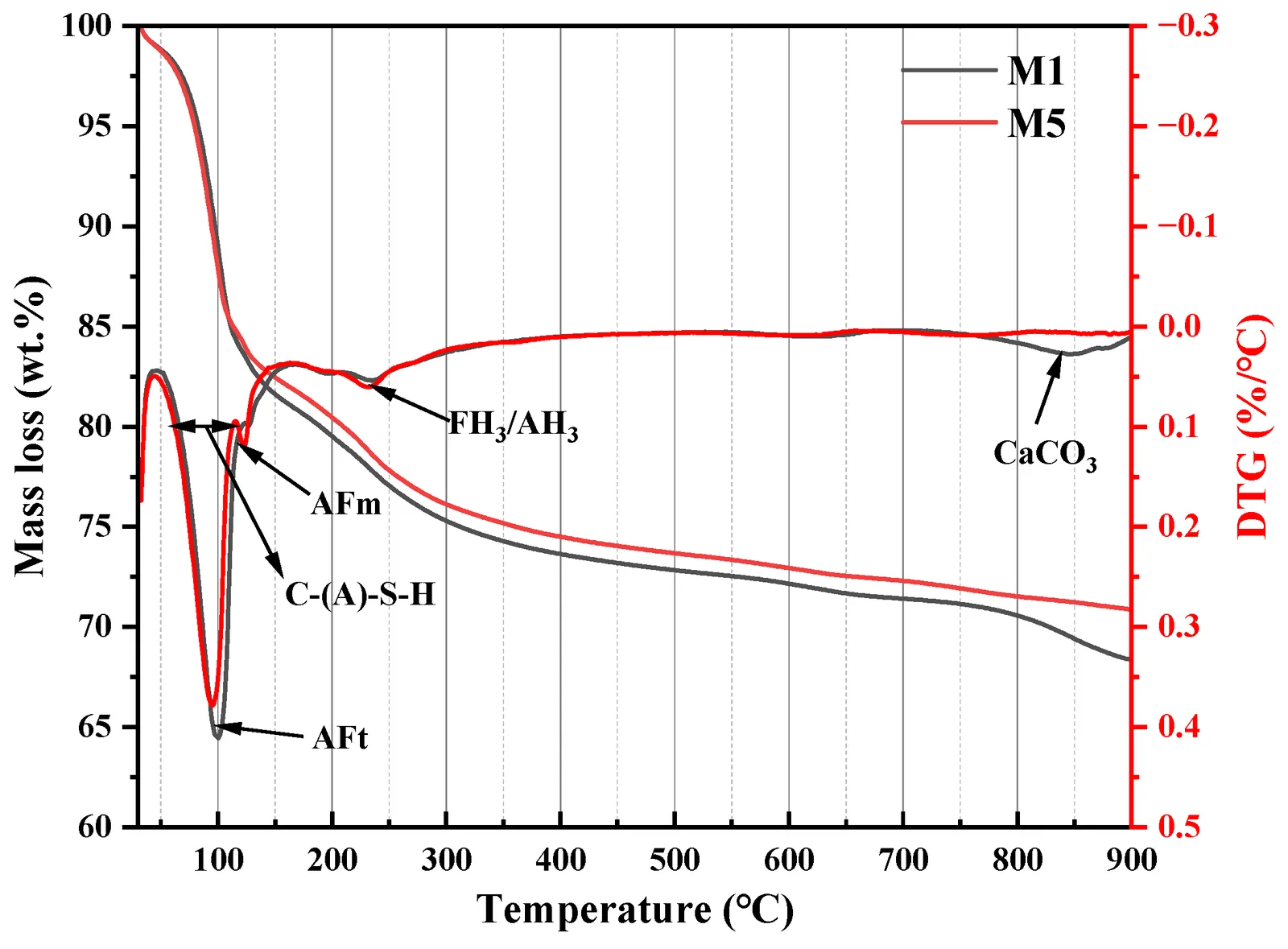
TG-DTG curves of SWFAC sample cured for 28 d.

Figure 15 shows the TG-DTG curves of M1 and M5 samples after 28 d of hydration, and the corresponding mass losses at different temperature ranges are summarized in Table 5. Three main decomposition regions were identified from the DTG curves. The mass loss below 200 °C was mainly attributed to the dehydration of AFt, AFm, C-(A)-S-H gels, and aluminum hydroxide-containing phases. The 200–350 °C region was associated with the dehydration of AFm and FH_3_/AH_3_ phases, while the higher-temperature region mainly corresponded to the decomposition of carbonate-containing phases. The mass losses of M1 and M5 in the range of 25–200 °C were 20.52 wt.% and 19.58 wt.%, respectively, indicating that M1 contained slightly more low-temperature dehydration products. This result agrees with the XRD and hydration calorimetry results, where M1 exhibited stronger AFt/AH_3_ signals and higher cumulative heat release, suggesting a higher hydration degree. In the 200–350 °C range, the mass losses of M1 and M5 were comparable (5.28 wt.% and 5.23 wt.%), indicating similar amounts of AFm and hydroxide-related products. For the 350–850 °C region, M1 showed a higher mass loss than M5 (4.88 wt.% and 3.94 wt.%), suggesting a slightly higher accumulation of high-temperature decomposable products. Although the increased HIRM content reduced the proportion of reactive C_4_A_3_$ and inhibited early hydration, M5 maintained continuous hydration at later ages.

#### 3.3.3. Micromorphology

**Figure 16:**
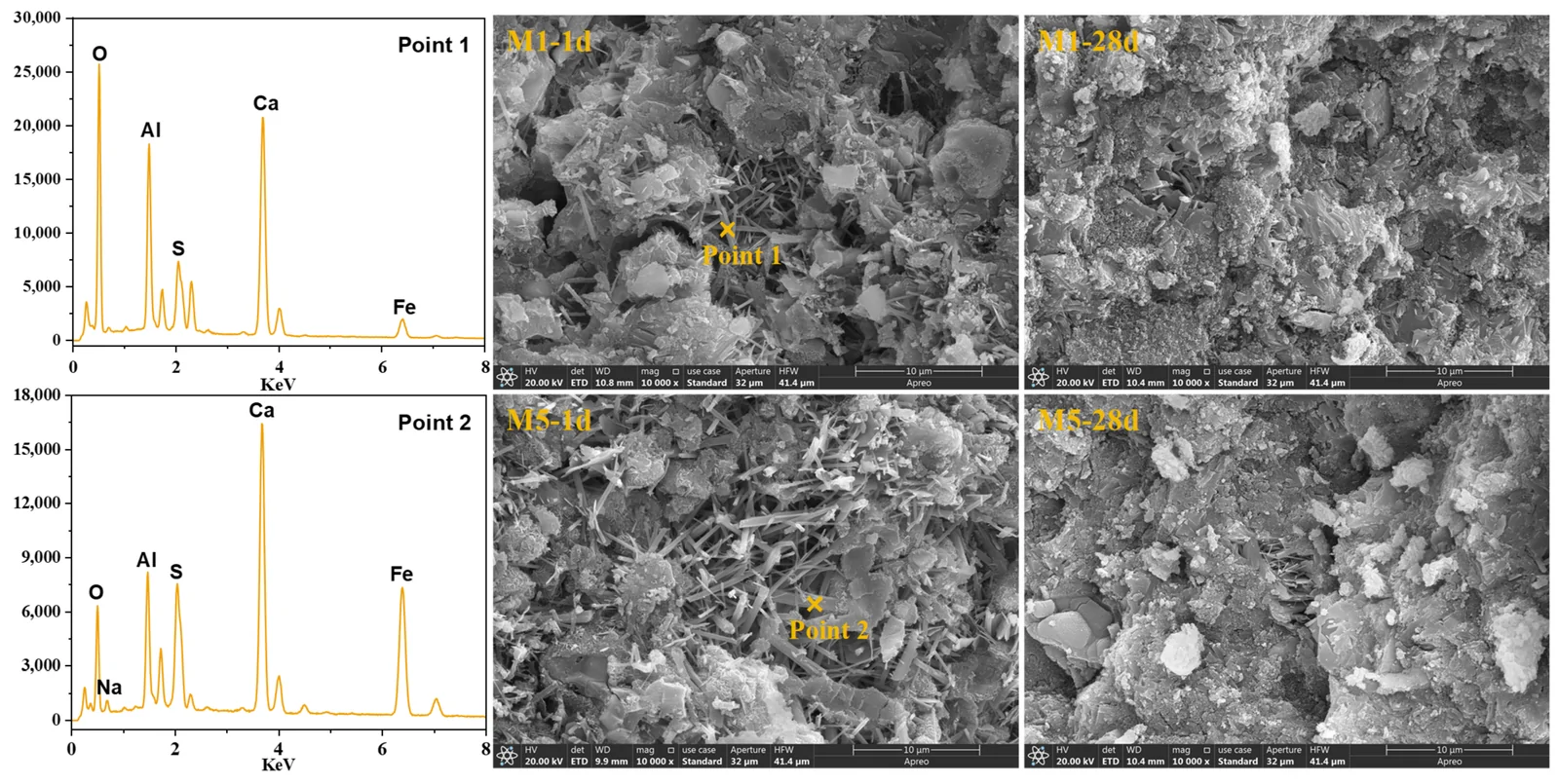
Microscopic morphology of SWFAC sample matrix at different hydration ages.

Figure 16 presents the microstructural evolution of SWFAC at different hydration ages. Significant differences in the microstructure of the SWFAC matrix were observed at different hydration stages. At 1 d (M1-1d and M5-1d), a certain amount of needle-like or rod-like ettringite crystals (Point 1) formed in both systems. However, the M5 sample, which contained a higher iron content, exhibited fewer hydration products and a more dispersed distribution of ettringite crystals (Point 2). A larger number of pores remained between particles, resulting in a relatively loose microstructure. This indicates that the M5 system experienced a lower hydration degree and a slower early hydration rate. After 28 d of hydration (M1-28d and M5-28d), large amounts of plate-like, flocculent, and gel-like hydration products were generated in both samples. The original pores were substantially filled, leading to a markedly denser matrix structure. Meanwhile, the microstructural differences between M1 and M5 became much less pronounced at 28 d, and both samples exhibited similar compactness and hydration product distributions. These observations indicate that the high-iron system was able to sustain hydration during the later stages and continuously generate hydration products, thereby compensating for its relatively insufficient early hydration and ultimately forming a dense and stable microstructure.

## 4. Conclusions

In this study, a low-carbon solid waste-based ferroaluminate cement was prepared from high-iron red mud and multi-industrial wastes, and the effects of mineral regulation and hydration behavior on performance development were systematically investigated. The main conclusions are summarized as follows:

(1) The synergistic reactions among CaO, Al_2_O_3_, Fe_2_O_3_, SiO_2_, and SO_3_ in multi-industrial wastes promoted the formation of C_4_A_3_$, C_2_S, and C_4_AF. The clinker mineral composition was effectively regulated by optimizing mix proportions and calcination parameters. Under the optimal condition of 1300 °C for 30 min, the clinker exhibited a balanced mineral assemblage containing 35.54 wt.% C_4_A_3_$, 37.86 wt.% C_2_S, and 15.56 wt.% C_4_AF, resulting in a 1 d and 28 d compressive strength of 40.0 MPa and 56.4 MPa, respectively.
(2) Increasing the content of HIRM promoted the formation of Fe-bearing clinker phases, especially C_4_AF, while slightly reducing the proportion of highly reactive C_4_A_3_$. As a result, the early hydration rate and heat release were moderately decreased. However, the Fe introduced by HIRM actively participated in hydration reactions and was incorporated into ettringite structures to form Fe-containing ettringite, indicating its important role in regulating hydration behavior and structural development.
(3) Hydration mechanism investigations revealed that the formation of AFt, AFm, AH_3_, Fe-AFt, and C-S-H-related hydration products promoted continuous pore filling and microstructural densification. The progressive accumulation of hydration products contributed to the development and long-term enhancement of compressive strength, enabling the SWFAC system to achieve a balance between early-age reactivity and later-age structural stability.

## Figures and Tables

**Table 1:** Chemical components and loss on ignition of raw materials (wt.%) [33].

Compound	CaO	SiO_2_	Al_2_O_3_	Fe_2_O_3_	SO_3_	MgO	Na_2_O	TiO_2_	LOI (%)
HIRM	2.84	5.72	23.68	57.78	0.39	0.17	2.73	5.25	11.30
CFBFA	16.37	37.48	29.42	4.38	8.63	0.96	0.24	1.21	3.60
DSA	58.49	0.51	0.28	0.82	34.75	0.66	0.17	0.04	18.30
CS	92.20	2.97	1.63	1.82	0.33	0.29	0.10	0.19	24.60
BR	0.40	15.01	75.09	1.59	0.02	—	0.13	5.08	0.40

**Table 2:** Mix design for the optimization of calcination conditions.

Sample	Raw Meal Mix Ratio (wt.%)						Calcination Condition	
	HIRM	CFBFA	DSA	CS	BR	Al_2_O_3_	C_Tem/°C	C_Time/min
T1	15	5	25	40	15	0	1000–1300	30–60
T2	15	5	25	40	0	15	1000–1300	30–60

**Table 3:** Mix design for mineral phase and performance optimization.

Sample	Raw Meal Mix Ratio (wt.%)					Calcination Conditions	
	HIRM	CFBFA	DSA	CS	BR	C_Tem/°C	C_Time/min
M1	10	5	27	38	20	1300	30
M2	10	10	30	35	15	1300	30
M3	15	5	27	38	15	1300	30
M4	20	3	27	37	13	1300	30
M5	25	2	25	37	11	1300	30
M6	30	2	23	37	8	1300	30

**Table 4:** Mineral phase composition of cement clinker obtained from different raw material ratios.

Sample	Mass Fraction (wt.%)				Rwp
	C_4_A_3_$	C_2_S	C_4_AF	f-CaO	
**M1**	35.54	37.86	15.56	1.11	9.45
**M2**	27.63	43.44	18.31	2.45	10.41
**M3**	34.43	36.59	22.57	2.73	8.93
**M5**	25.77	33.19	26.71	3.11	11.59

**Table 5:** Mass loss of SWFAC samples cured for 28 d at different temperature stages.

Samples	Different Temperature Stages (wt.%)		
	25–200 °C	200–350 °C	350–850 °C
M1	20.52	5.28	4.88
M5	19.58	5.23	3.94

## Data Availability

The original contributions presented in this study are included in the article. Further inquiries can be directed to the corresponding authors.
